# Effect of oxidative stress on heme oxygenase-1 expression in patients with gestational diabetes mellitus

**DOI:** 10.3892/etm.2013.1435

**Published:** 2013-12-04

**Authors:** GANG XIN, JUAN DU, YONG-TAO WANG, TING-TING LIANG

**Affiliations:** 1 Department of Obstetrics and Gynecology, The Second Hospital of Shandong University, Jinan, Shandong 250033, P.R. China; 2 Department of Obstetrisc, Women and Children’s Hospital of Jinan, Jinan, Shandong 250001, P.R. China; 3 Central Laboratory, The Second Hospital of Shandong University, Jinan, Shandong 250033, P.R. China

**Keywords:** anti-oxidative stress, heme oxygenase-1, gestational diabetes mellitus

## Abstract

The anti-oxidative stress effect of heme oxygenase-1 (HO-1) expression is being increasingly studied. However, few studies regarding HO-1 have been conducted in patients with gestational diabetes mellitus (GDM). In the present study, HO-1 expression was compared in peripheral blood mononuclear cells at 24–28 weeks of pregnancy in patients with GDM and healthy females, to investigate the correlation between HO-1 and oxidative stress by calculation of MDA content in the peripheral blood serum (thiobarbituric acid method), tested ROS (flow cytometry method), HO-1mRNA (RT-PCR method), and HO-1 protein (western blotting method) of Mononuclear cells. The results show that the levels of serum malonaldehyde (MDA), reactive oxygen species (ROS), *HO-1* mRNA and HO-1 protein expression in peripheral blood mononuclear cells were higher in the GDM group than in the control group. Correlation analysis showed that the expression levels of HO-1 protein were positively correlated with the *HO-1* mRNA expression levels (r=0.680; P=0.000), and the levels of ROS (r=0.572; P=0.000) and MDA (r=0.614; P=0.000). *HO-1* mRNA expression levels were found to positively correlate with the levels of MDA (r=0.451; P=0.010) and fasting plasma glucose (FPG; r=0.337; P=0.039). Partial correlation analysis demonstrated that, after removing the effects of body mass index, FPG and 2-h plasma glucose, HO-1 protein expression levels were positively correlated with the levels of *HO-1* mRNA expression (r=0.611; P=0.005), ROS (r=0.526; P=0.021) and MDA (r=0.519; P=0.015). These findings indicate that pregnant females with GDM may be protected against oxidative injury due to the induction of adaptive and compensatory expression of HO-1 to guard against oxidative stress induced by high glucose levels.

## Introduction

Gestational diabetes mellitus (GDM) is defined as any degree of glucose intolerance with onset or first recognition during pregnancy. GDM prevalence has increased by 16–127% in several ethnicity groups during the past 20 years (1). GDM has become a significant cause of maternal and neonatal morbidity and mortality resulting from macrosomia, fetal distress, fetal death, respiratory distress syndrome, hypoglycemia, hyperbilirubinemia, polycythemia and hypocalcemia. Clinicians and epidemiologists have identified that females who experience GDM are at a greater risk of developing type II diabetes (2,3). A recent study suggested that the cumulative incidence rate of postpartum type II diabetes in patients with GDM is 50–70% in China (4). According to World Health Organization guidelines (5), as a special diabetes type, GDM is a high-risk factor for diabetes, particularly in young females. In addition, GDM has been hypothesized to be an early stage in the development of type II diabetes. Ceriello discussed the ‘common soil’ theory, suggesting that oxidative stress represents the common factor in insulin resistance, diabetes and cardiovascular disease (6). Malonaldehyde (MDA) and reactive oxygen species (ROS) levels reflect the degree of oxidative injury. Oxidative injury can lead to the upregulation of protective antioxidant genes, including superoxide dismutase, glutathione peroxidase, thioredoxin reductase and heme oxygenase-1 (HO-1). The anti-oxidative stress effect of HO-1 is being increasingly investigated (7,8). The *HO-1* gene, located on human chromosome 22q12, HO-1 protein is also widely distributed in microsomes of various tissues in mammalian animals. HO-1 is normally expressed at low levels; however, oxidative stress can induce an increased level of expression and activity (4,9,10). The cell-protection effect of HO-1 has become a key topic of investigation in organ transplantation, ischemia-reperfusion injury, cardiovascular and cerebrovascular disease, bronchial asthma and preeclampsia (11). A previous study investigated the effect of the serum concentration and total activity of HO-1 on neovascularization, and demonstrated that upregulated expression of the HO-1 gene and increased HO-1 activity exerted a protective effect against the development of diabetic complications (12). Animal experiments have demonstrated that the expression levels of HO-1 in pancreatic islet cells were elevated by exposure to high glucose levels (13).

At present, studies on oxidative stress in diabetes mainly focus on type II diabetes and its complications. It has been hypothesized that oxidative injury appears in the early stages of diabetes (impaired glucose tolerance) (13). However, there are few studies describing oxidative stress and the related cellular protective response in patients with GDM. In the present study, HO-1 expression was compared in peripheral blood mononuclear cells from patients at 24–28 weeks of pregnancy with GDM and healthy females, to investigate the correlation between HO-1 and oxidative stress.

## Materials and methods

### Patients

This case-control study was approved by the Ethics Committee of The Second Hospital of Shandong University (Jinan, China). Written consent was obtained from all participants. Patients with GDM (GDM group) and healthy pregnant females (control group) at 24–28 weeks of pregnancy from Jinan Maternal and Child Health Hospital (Jinan, China) and the Department of Obstetrics and Gynecology (The Second Hospital of Shandong University), were included in this study, which was conducted between May 2009 and November 2011. The American Diabetes Association (ADA) 2004 diagnostic criteria were used (14). The pregnant females were screened for GDM at 24–28 weeks of pregnancy using the 1-h oral glucose challenge test with 50 g glucose. The patients who failed this screening test (glucose, ≥7.8 mmol/l or 140 mg/dl) were then followed up within one to two weeks with 3-h oral glucose tolerance tests (OGTTs) using 100 g glucose. Patients were diagnosed with GDM if two or more of the 100 g OGTT glucose levels exceeded the following thresholds based on the ADA criteria: fasting plasma glucose (FPG) (≥95 mg/dl); 1-h, ≥10 mmol/l (≥180 mg/dl); 2-h, ≥8.6 mmol/l (≥155 mg/dl); and 3-h, ≥7.8 mmol/l (≥140 mg/dl) (14).

**Figure 1:**
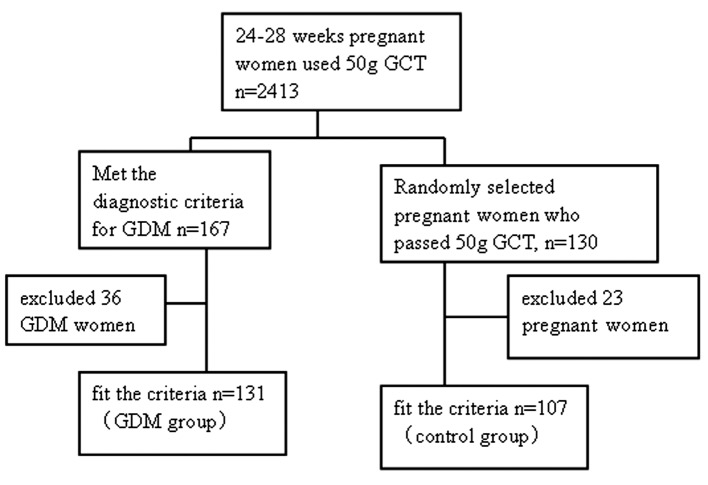
Patient selection.

The following inclusion criteria were used: 20–35 years of age, regular menstrual cycle, single fetus, normal fetal head position, normal liver and kidney function and normal routine blood and urine tests (with the exception of urine glucose). The exclusion criteria were as follows: Smoking and alcohol consumption in any amount during the pregnancy, current fever, complications due to pregnancy or other medical complications, and current intake of any amount of vitamin E, vitamin C and/or antibiotics during the pregnancy (Fig. 1).

### Materials

Human monocyte isolation liquid and Percoll cell separation medium were purchased from Pharmacia Corporation (St. Louis, MO, USA). cDNA and β-actin primers were obtained from Shanghai Yingjun Biotechnology Co., Ltd. (Shanghai, China). Rabbit anti-human HO-1 antibody (SC-10789) and goat anti-human HO-1 polyclonal antibody (SC-34674) were purchased from Santa Cruz Biotechnology, Inc. (Santa Cruz, CA, USA). FITC-labeled goat anti-rabbit IgG antibody, MDA and ROS assay kits were obtained from Zhongshan Golden Bridge Biotech Company (Beijing, China), Jiancheng Biological Engineering Institute (Jiangsu, China) and Biyuntian Biotechnology Company (Jiangsu, China), respectively. Goat β-actin polyclonal antibody was purchased from Boshide Biological Engineering Co., Ltd., (Wuhan, China) and polyvinylidene fluoride membranes (PVDF) were purchased from Biyuntian Biotechnology Co., Ltd. (Shanghai, China).

The *HO-1* and *β-actin* sequences were obtained from GenBank. Primers were designed using the Vector NTI software (Invitrogen Life Technologies, Carlsbad, CA, USA) and the sequences were as follows: *HO-1* forward, 5′-ctggaggaggagattgagcg-3′ and reverse, 5′-taaggaccatcggagaagcg-3′ (fragment size, 637 bp; T_m_, 53°C); *β-actin* forward, 5′-agcgagcatcccccaaagtt-3′ and reverse, 5′-gggcacgaaggctcatcatt-3′ (fragment size, 285 bp; T_m_, 52°C).

### Isolation, purification and culture of human peripheral blood mononuclear cells

Human peripheral blood (10 ml) was collected from an elbow vein and treated with lymphocyte separation medium, consisting of Ficoll-Hypaque (Pharmacia Corporation), to obtain the human mononuclear cell suspension. Percoll was used to separate the human mononuclear cells. RPMI-1640 medium was utilized to adjust the mononuclear cell concentration to 4×10^5^ cells/l. The sample was incubated at 37°C with 5% CO_2_ for 2 h.

### ROS assay

Mononuclear cells were collected and suspended in diluted dichloro-dihydro-fluorescein diacetate solution (Sigma-Aldrich, St. Louis, MO, USA) at a density of 1×10^6^ cells/ml and incubated at 37°C for 20 min. The cells were washed two to three times with RPMI-1640 medium and analyzed with flow cytometry (Becton Dickinson & Co., Franklin Lakes, NJ, USA). The excitation and emission wavelengths were set to 488 and 525 nm, respectively.

### MDA assay

The thiobarbituric acid method was used to determine the levels of MDA in the cell supernatants according to the manufacturer’s instructions. Tetraethoxypropane (10 nmol/ml; 0.6 ml) was used as a standard sample, distilled water (2 ml) was used as a blank sample, the cell supernatants (0.6 ml) were used as test samples. A spectrophotometer (Hitachi, Tokyo, Japan) was used to determine the absorbance values of each tube. The MDA content in the medium was calculated using the following formula: MDA (nmol/ml) = (test tube absorbance - blank tube absorbance)/(standard tube absorbance - standard blank tube absorbance).

### Analysis of HO-1 mRNA expression by reverse transcription polymerase chain reaction (RT-PCR)

Total cellular RNA was extracted from collected mononuclear cells to measure the RNA concentration and purity at 260- and 280-nm absorbance. The total RNA was reverse transcribed to produce cDNA, which was amplified. The PCR products were detected by agarose gel electrophoresis and analyzed using the UVIB and gel image analysis system (Jiapeng technology Co., Ltd., Shanghai, China). *HO-1* mRNA expression in the GDM and control groups was compared. The *HO-1* products of the two groups were detected by agarose gel electrophoresis and analyzed using the Image-Pro Plus 3 image analysis software (Media Cybernetics, Inc., Warrendale, PA, USA). The gene expression values were calculated according to the following equation: Gene expression values (%) = [each gene band area × (band brightness - background brightness)]/[*β-actin* gene band area × (band brightness - background brightness)] × 100.

### Analysis of HO-1 protein expression by western blotting

Following separation and purification, human peripheral blood mononuclear cells were centrifuged at 3,000 rpm (Allegra X-15R; Beckman Coulter, Inc., Brea, CA, USA) for 5 min and stored at −70°C for 4 h for cell lysis. The sample was dissolved in 1 ml of SDS buffer, placed in a boiling water bath for 10 min, centrifuged at 12,000 rpm for 5 min and stored at −20°C. Discontinuous SDS-PAGE was carried out at 80 V for 30 min, and then at 110 V for 2 h. At 100 V and at room temperature, wet transfer was used to transfer the target protein (HO-1) and the internal control (β-actin) onto PVDF membranes for 2 h. Following blocking and washing steps, blots were incubated with goat polyclonal anti-HO-1 and anti-β-actin antibodies diluted to 1:800 in PBS buffer with 0.05% Tween-20 at room temperature for 2 h. The blots were washed and incubated with horseradish peroxidase-labeled rabbit anti-goat IgG diluted to 1:5,000 in PBS buffer with 0.05% Tween-20 at room temperature for 2 h. Following color development with 3,3′-diaminobenzidine, the membranes were dried and images were captured. Image-Pro Plus image analysis software was utilized to analyze the protein bands. Expression of the HO-1 protein (%) = the gray value of target protein band/the gray value of the internal control protein band × 100.

### Statistical analysis

Statistical analysis was performed using SPSS software v13.0 (SPSS, Inc., Chicago, IL, USA). Quantitative data are expressed as the mean ± standard deviation. Student’s t-tests were performed for comparison of the two groups. Pearson correlation was used to analyze the normally distributed data, while rank correlation analysis was performed with further partial correlation analysis for the data with non-normal distribution. P<0.05 was considered to indicate a statistically significant difference.

## Results

### General information

No significant difference was identified in mean age between the GDM and control groups. The body mass index (BMI), fasting plasma glucose (FPG) and OGTT (1- and 2-h) measurements were significantly higher in the GDM group compared with those in the control group (Table I).

### Oxidative stress parameters

The serum MDA and ROS levels in peripheral blood mononuclear cells were significantly higher in the GDM group compared with those in the control group (P<0.01; Table II).

### HO-1 mRNA expression (RT-PCR)

**Figure 2:**
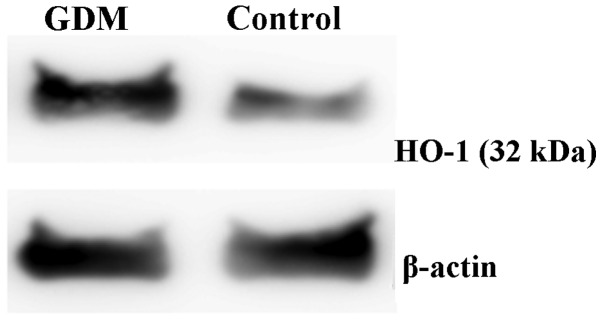
Expression of *HO-1* mRNA, as detected by reverse transcription polymerase chain reaction. *HO-1* mRNA expression in peripheral blood mononuclear cells from the GDM group was higher than that in peripheral blood mononuclear cells from the control group. GDM, gestational diabetes mellitus; HO-1, heme oxygenase-1.

Gene amplification bands for *HO-1* and internal control *β-actin* of the expected sizes (637 and 285 bp, respectively) were identified in the GDM and control groups. The *HO-1* mRNA expression levels in the peripheral blood mononuclear cells from the GDM group were significantly higher than those in the peripheral blood mononuclear cells from the control group (Fig. 2; Table III).

### HO-1 protein expression (western blotting)

**Figure 3:**
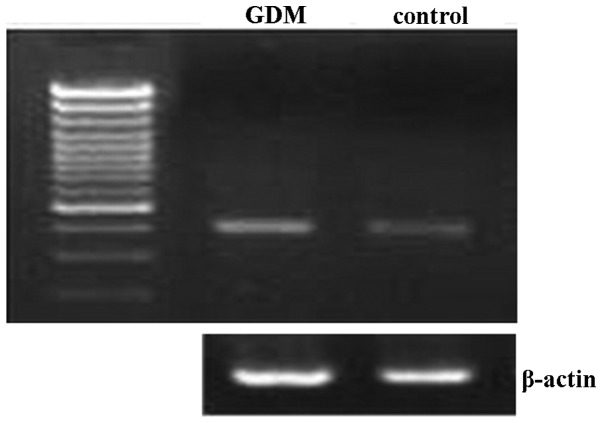
Western blot analysis of the expression of HO-1 (32 kDa) and the reference β-actin protein in the two groups. The results indicate that HO-1 protein expression in peripheral blood mononuclear cells from the GDM group was higher than that in peripheral blood mononuclear cells from the control group. GDM, gestational diabetes mellitus; HO-1, heme oxygenase-1.

HO-1 (32 kDa) and internal control β-actin protein-expression bands, identified via antibody staining, were examined in the GDM and control groups. HO-1 protein expression in the peripheral blood mononuclear cells from the GDM group was significantly higher than that in the peripheral blood mononuclear cells from the control group (Fig. 3; Table III).

### Correlation between oxidative stress parameters and HO-1 expression

Correlation analysis indicated that the expression levels of the HO-1 protein were positively correlated with the levels of *HO-1* mRNA expression (r=0.680; P=0.000), ROS (r=0.572; P=0.000) and MDA (r=0.614; P=0.000). The *HO-1* mRNA expression was positively correlated with the levels of MDA (r=0.451; P= 0.010) and FPG (r=0.337; P=0.039). There was a significant positive correlation between the levels of MDA and ROS (r=0.695; P=0.000).

Partial correlation analysis demonstrated that, after removing the effects of BMI, FPG and 2-h plasma glucose, HO-1 protein expression was positively correlated with the levels of *HO-1* mRNA expression (r=0.611; P=0.005), ROS (r=0.526; P=0.021) and MDA (r=0.519; P=0.015). MDA levels were significantly positively correlated with ROS levels (r=0.734; P=0.000).

## Discussion

Oxidative stress-induced hyperglycemia is considered a key step in the pathogenesis of chronic diabetes complications. Hyperglycemia causing an increased cellular ROS level and anti-oxidative dysfunction leads to cellular oxidative stress. It has been reported that oxidative stress is also important in GDM (15,16). Biri *et al* reported that the antioxidant system was impaired and that the superoxide dismutase activity level was increased in the cord blood and placental tissue of patients with GDM (17). Chen and Scholl demonstrated that MDA levels were increased, antioxidant enzyme activity was decreased and the blood sugar levels were positively correlated with the concentration of MDA in patients with GDM (18).

HO-1 is an inducible isoform of HO that is produced in response to oxidative stress and is regulated by a single transcription factor (nuclear factor E2 p45-related factor 2). HO, the only rate-limiting enzyme involved in heme degradation, converts heme into carbon monoxide, iron and biliverdin, which is followed by the conversion of biliverdin to bilirubin by biliverdin reductase. The anti-oxidative, anti-inflammatory, anti-apoptosis, signaling and immune regulatory effects of the aforementioned metabolites enable HO-1 to provide extensive tissue protection. The role of HO-1 in organ transplantation, ischemia reperfusion injury, cardiovascular and cerebrovascular disease, bronchial asthma and preeclampsia is being increasingly investigated (19–21).

A previous study investigated the effect of the serum concentration and total activity of HO-1 on neovascularization, and demonstrated that upregulated expression levels of the HO-1 gene and increased HO-1 activity had a protective effect against the development of diabetic complications (13). Animal experiments demonstrated that the HO-1 expression levels in pancreatic islet cells was elevated by exposure to high glucose levels (22). In another experiment, mice undergoing a partial pancreatectomy had elevated blood glucose levels and subsequently lower levels of *HO-1* gene expression in pancreatic islet cells. Elevated HO-1 levels in circulating plasma have been identified in Chinese patients who have impaired glucose tolerance (23) and type II diabetes (24).

Studies using human umbilical vein endothelial cells have demonstrated that increased HO-1 expression and activity, induced by high glucose levels, was involved in oxidative DNA and protein damage, as well as increased expression levels of vasoactive factors (25,26). A previous study using euglycemic-hyperinsulinemic glucose clamps in patients with type II diabetes demonstrated that in the skeletal muscle of the diabetes group, the *HO-1* mRNA expression levels were 55% lower than those in the control group prior to clamp placement; however, the levels increased by 70-fold following clamp placement. By contrast, in the control group, the *HO-1* mRNA expression levels were not elevated following clamp placement (27). Another study demonstrated that in patients with type II diabetes accompanied by kidney disease, various oxidative parameters were significantly increased, and the severity of renal failure was positively correlated with *HO-1* expression (28).

There are a limited number of studies describing HO-1 expression levels in GDM as an early stage of type II diabetes. Qiu *et al*(29) found that median serum HO-1 concentrations in early pregnancy were lower in patients who subsequently developed GDM compared with those who did not. Following adjustment for maternal age, race, family history of type II diabetes mellitus and prepregnancy BMI, patients with HO-1 levels ≥3.05 ng/ml (highest decile) experienced a 74% reduction in GDM risk (95% CI, 0.09–0.77) compared with patients whose HO-1 concentrations were <1.23 ng/ml. The serum HO-1 concentration was negatively associated with subsequent GDM risk.

The key findings of the present study are as follows: i) ROS and serum MDA levels in the peripheral blood mononuclear cells are significantly higher in females with GDM than healthy individuals; and ii) the expression of HO-1 in peripheral blood mononuclear cells was significantly lower in healthy pregnant females than in patients with GDM. As previously described, high glucose levels increased the expression and activity level of HO-1 (27). In order to exclude the effects of BMI and blood sugar, partial correlation analysis was performed after controlling for the aforementioned factors. It was identified that the expression of HO-1 was correlated with the levels of ROS, indicating that ROS may induce the expression of HO-1 and the enhancement of its activity. The limitation of the present study is the lack of data regarding the possible effects of insulin resistance, considering the abnormal glucose tolerance and insulin resistance observed in GMD patients. In addition, the correlation between oxidative stress and HO-1 expression was only investigated during a 24- to 28-week period; additional data should be collected for analysis during late pregnancy and childbirth.

In conclusion, the results of the present study indicate that pregnant females with GDM may be protected against oxidative injury by the induction of adaptive and compensatory increased expression of HO-1 to protect against oxidative stress induced by high glucose levels.

## Figures and Tables

**Table I:** General information.

Parameter	GDM (n=131)	Control (n=107)	P-value
Age, years	25.13±5.27	25.09±6.05	0.9566
BMI	24.15±3.51	22.06±5.22	0.0069
FPG, mmol/l	6.03±4.37	4.69±3.22	0.0089
1-h OGTT, mmol/l	10.42±3.28	7.61±1.09	0.0000
2-h OGTT, mmol/l	9.04±1.63	6.68±2.15	0.0027

GDM, gestational diabetes mellitus; BMI, body mass index; FPG, fasting plasma glucose; OGTT, oral glucose tolerance test.

**Table II:** Comparison of oxidative stress parameters between the two groups.

Parameter	GDM (n=131)	Control (n=107)	P-value
ROS (MFI)	97.51±26.27	67.42±21.36	<0.0500
MDA (nmol/ml)	39.83±13.24	31.55±14.41	<0.0500

GDM, gestational diabetes mellitus; ROS, reactive oxygen species; MFI; mean fluorescence intensity; MDA, malonaldehyde.

**Table III:** mRNA and protein expression of HO-1 in peripheral blood mononuclear cells in the two groups.

Parameter	GDM (n=131)	Control (n=107)	P-value
*HO-1/β-actin*			
mRNA, %	0.55±0.31	0.32±0.19	<0.0500
HO-1 protein, %	63.24±12.43	18.26±13.28	<0.0500

GDM, gestational diabetes mellitus; HO-1, heme oxygenase-1.
